# Supplementary material for: Exploring the pharmacist’s role in supporting newcomer international students and their families with the transition to the Canadian healthcare system including medication use: Protocol for a qualitative study
Source: PLoS One. 2024 Jun 6;19(6):e0304780. doi: 10.1371/journal.pone.0304780 (PMC11156380; doi:10.1371/journal.pone.0304780)
Supplement: S1 Appendix — (PDF) [file pone.0304780.s001.pdf]

S1 Appendix – Interview guide

Demographic data:

- Name:
- Age:
- Gender:
- Date of arrival to Canada:
- Living alone or with family/partner:

Instructions:

The interview will be recorded and transcribed using Microsoft Teams. You have the right to turn off your camera or stop the recording at any time during the interview if you feel uncomfortable, skip any question, or withdraw from the interview. However, recording the interview is not optional. If you choose to stop or refuse to record, then you will be withdrawn from the study.

<u>List of questions:</u>

1. **Could you please tell me about yourself and your program of study?**

   Probes:
   - What led you to pursue your current program of study?
   - Have you had any opportunities to network with new people or make new friends?
   - How did it feel to leave your country of origin and move to Canada?
2. **I'm mainly interested in transitioning to the Canadian healthcare system by international students and their families, could you please tell me more about your experience?**

   Probes:
   - What are the main similarities and differences between the healthcare system here and in your country of origin?
   - What are the main challenges you faced during your initial contact with the Canadian healthcare system? And from your perspective, what are the causes of these challenges?
   - Could you please tell me about the impact of such challenges on your everyday life?
3. **Could you please tell me about your experience with medication changes/ disease management when you moved to Canada? And the main barriers you faced? What went well?**

Probes:

- Do you or any member of your family have a chronic disease or regularly use certain medications, vitamins, food supplements, or herbal products? If yes, could you please tell me more about it?
- Have you used any pharmacy services or healthcare services that helped you with your medications? If yes, how do you describe your satisfaction with these services?
- In your opinion, what are the main differences between the pharmacist's role here versus their role in your country of origin?

4. **What actions would you consider helpful in supporting international students with regard to a continuous and effective transition to the healthcare system here in Canada?**

Probes:

- How can the pharmacist assist international students and their families with transitioning to the Canadian healthcare system? more specifically in addressing medication-related issues?
- To better assist international students and their families, what can be done to improve pharmaceutical care services?

5. **Could you please tell me about your experience with using virtual care[1]?**

Probes: if they have experience with using virtual care:

- What are the pros and cons of using in-person versus virtual pharmaceutical services?
- What do you think of using virtual care to deliver pharmaceutical services to help international students and their families transition to the Canadian healthcare system?
- What are your suggestions to improve virtual care services?

[1] According to the Canadian Institute for Health Information, virtual care has been defined as "any interaction between patients and/or members of their circle of care, occurring remotely, using any forms of communication or information technologies, with the aim of facilitating or maximizing the quality and effectiveness of patient care".
