# Supplementary material for: Exploring the pharmacist’s role in supporting newcomer international students and their families with the transition to the Canadian healthcare system including medication use: Protocol for a qualitative study
Source: PLoS One. 2024 Jun 6;19(6):e0304780. doi: 10.1371/journal.pone.0304780 (PMC11156380; doi:10.1371/journal.pone.0304780)
Supplement: S2 Appendix — (PDF) [file pone.0304780.s002.pdf]

S2 Appendix – Consolidated criteria for reporting qualitative studies (COREQ): 32-item checklist

No	Item	Guide questions/description	Answer
**Domain 1: Research team and reflexivity**			
Personal Characteristics			
1	Interviewer/facilitator	Which author/s conducted the interview or focus group?	Y. Aboelzahab
2	Credentials	What were the researcher's credentials? E.g. PhD, MD	BScPhm MHSc
3	Occupation	What was their occupation at the time of the study?	PhD Student
4	Gender	Was the researcher male or female?	Female
5	Experience and training	What experience or training did the researcher have?	3+ years of conducting qualitative research including master's qualitative study and taking several graduate qualitative courses and workshops in qualitative research
Relationship with participants			
6	Relationship established	Was a relationship established prior to study commencement?	No
7	Participant knowledge of the interviewer	What did the participants know about the researcher? e.g. personal goals, reasons for doing the research	Reasons for doing the study: to help establish a foundational block to help support newcomer international students and their families with their transition to the Canadian healthcare system
8	Interviewer characteristics	What characteristics were reported about the interviewer/facilitator?	N/A at the current study stage

		e.g. Bias, assumptions, reasons and interests in the research topic	
**Domain 2: Study design**			
Theoretical framework			
9	Methodological orientation and Theory	What methodological orientation was stated to underpin the study? e.g. grounded theory, discourse analysis, ethnography, phenomenology, content analysis	Exploratory descriptive methodology
Participant selection			
10	Sampling	How were participants selected? e.g. purposive, convenience, consecutive, snowball	Purposeful sampling
11	Method of approach	How were participants approached? e.g. face-to-face, telephone, mail, email	Face-to-face, email, social media through study poster
12	Sample size	How many participants were in the study?	Approximately 20
13	Non-participation	How many people refused to participate or dropped out? Reasons?	N/A at the current study stage. Reporting will occur after data collection has concluded.
Setting			
14	Setting of data collection	Where was the data collected? e.g. home, clinic, workplace	Workplace at Leslie Dan Faculty of Pharmacy, University of Toronto and home office
15	Presence of nonparticipants	Was anyone else present besides the participants and researchers?	No
16	Description of sample	What are the important characteristics of the sample? e.g.	Demographic data

		demographic data, date	
Data collection			
17	Interview guide	Were questions, prompts, guides provided by the authors? Was it pilot tested?	Yes
18	Repeat interviews	Were repeat interviews carried out? If yes, how many?	N/A at the current study stage
19	Audio/visual recording	Did the research use audio or visual recording to collect the data?	Yes
20	Field notes	Were field notes made during and/or after the interview or focus group?	N/A at the current study stage
21	Duration	What was the duration of the interviews or focus group?	30-60 minutes
22	Data saturation	Was data saturation discussed?	Yes
23	Transcripts returned	Were transcripts returned to participants for comment and/or correction?	N/A at the current study stage
**Domain 3: analysis and findings**			
Data analysis			
24	Number of data coders	How many data coders coded the data?	To be determined
25	Description of the coding tree	Did authors provide a description of the coding tree?	N/A at the current study stage
26	Derivation of themes	Were themes identified in advance or derived from the data?	Derived from the data
27	Software	What software, if applicable, was used to manage the data?	NVivo
28	Participant checking	Did participants provide feedback on the findings?	N/A at the current study stage
Reporting			

29	Quotations presented	Were participant quotations presented to illustrate the themes/findings? Was each quotation identified? e.g. participant number	N/A at the current study stage
30	Data and findings consistent	Was there consistency between the data presented and the findings?	N/A at the current study stage
31	Clarity of major themes	Were major themes clearly presented in the findings?	N/A at the current study stage
32	Clarity of minor themes	Is there a description of diverse cases or discussion of minor themes?	N/A at the current study stage
