# Supplementary material for: Exploring the pharmacist’s role in supporting newcomer international students and their families with the transition to the Canadian healthcare system including medication use: Protocol for a qualitative study
Source: PLoS One. 2024 Jun 6;19(6):e0304780. doi: 10.1371/journal.pone.0304780 (PMC11156380; doi:10.1371/journal.pone.0304780)
Supplement: Attachment — Submitted filename: Response to Reviewers.docx [file pone.0304780.s004.docx]

May 10, 2024

RE: Manuscript# PONE-D-24-06002

Dear Dr. Thapa,

Please find attached our revised manuscript entitled **“Exploring the pharmacist’s role in supporting newcomer international students and their families with the transition to the Canadian healthcare system including medication use: Protocol** **for a qualitative study**” which we are resubmitting for your consideration for publication in ***PLOS One*** Journal.

We thank you and the reviewers for the helpful comments on our manuscript and the opportunity to submit a revised version. We recognize there were questions that precluded publication. To address the comments, we have modified the text and provided additional explanations. We have detailed the changes below.

This work is supported by Leslie Dan Faculty of Pharmacy, University of Toronto, and the Network for Improving Health Systems (NIHS) Trainee Award, University of Toronto. There was no additional external funding received for this study.

Thank you for considering our work and please let us know if you have any further questions.

Sincerely,

Lisa Dolovich, BScPhm, PharmD, MSc

Professor and Dean

Leslie Dan Faculty of Pharmacy,

University of Toronto

Toronto, Ontario, Canada

lisa.dolovich@utoronto.ca

**Editor Comments:**

Thank you for stating in your Funding Statement:

“This work is supported by Leslie Dan Faculty of Pharmacy, University of Toronto, and the Network for Improving Health Systems (NIHS) Trainee Award, University of Toronto”

Please provide an amended statement that declares *all* the funding or sources of support (whether external or internal to your organization) received during this study, as detailed online in our guide for authors at http://journals.plos.org/plosone/s/submit-now. Please also include the statement “There was no additional external funding received for this study.” in your updated Funding Statement.

Updated Funding Statement: “This work is supported by Leslie Dan Faculty of Pharmacy, University of Toronto, and the Network for Improving Health Systems (NIHS) Trainee Award, University of Toronto. There was no additional external funding received for this study.”

Dear Authors,
I enjoyed reading your paper. The paper tries to explores the challenges faced by international students in Canada. However, the protocol can be equally important to other countries who are receiving international students. Therefore, I request you to add more relevant information in background, methodology and discussion section.

**Response to Editor**

We would like to thank the editor for this comment. In response, we added more relevant information to several sections of our manuscript as follows:

- Introduction section: *“**This study recognizes the increasing global mobility of international students and the crucial role healthcare systems play in ensuring their well-being and academic success[27]. As countries continue to attract students from diverse backgrounds, it is essential to understand and address the unique healthcare challenges faced by these students[9,27]. By exploring the healthcare experiences of newcomer international students in Canada, this research aims to illuminate the broader patterns and obstacles that could be prevalent across other host countries around the world.”*
- Methodology section: *“We will make concerted efforts to include international students from diverse countries. This diversity will enable the collection of data from students who have experienced various healthcare systems, providing a richer base of information for identifying common challenges* *and developing effective strategies to address them.”*
- Discussion section: *“Moreover, the insights from this study on the transition of newcomer international students to the Canadian healthcare system could be highly relevant to other countries with significant international student populations. Given the shared challenges these countries face, the findings and recommendations from this study can be adapted to enhance healthcare access for international students worldwide. The barriers to healthcare access encountered by international students are not unique to Canada but are potentially common across other host countries[9,27]. By identifying these challenges within the Canadian context, this study will provide valuable insights that could be tailored by other nations to address similar issues. Such adaptations are crucial for countries with large international student populations facing comparable healthcare access challenges. This approach can broaden our understanding of global healthcare needs for international students, highlighting both commonalities and variations.”*

**At this stage, I have following comments:**
Background should contain some of the information about global trends of students migration, how they face health care challenges, they provide an overview of Canada. Why it is important to have access to health care services, due to lack of which how is the situation in global, in regional and in Canada?

We added a section that briefly addresses global trends of students’ migration.

- Introduction section: *“A large portion of the newcomer population consists of international students[8]. The phenomenon of international student mobility is a global trend, driven by students' pursuit of educational and career opportunities across borders[8]. Each year, millions of students navigate complex and unfamiliar healthcare systems in their host countries, facing several barriers, and a general lack of knowledge about available healthcare resources[9–11]. Access to healthcare is crucial for international students, not only to prevent the exacerbation of health disparities but also to ensure their academic and social success. Without adequate healthcare services, international students are at a higher risk of facing untreated health issues, which can impede their ability to perform academically, integrate socially, and overall, succeed in their international educational endeavors [9].”*

Aims and objectives can be stated as a paragraph in the last portion of introduction section.

We added the study aims and objectives as a paragraph in the last portion of the introduction section: *“Our study aims to provide a comprehensive understanding of the unique lived experiences of international students. We will explore the healthcare needs of these students as they transition from healthcare systems in their countries of origin to Canada. Additionally, the study will identify the diverse challenges that these students face. It also seeks to uncover gaps in healthcare delivery that may hinder the continuity of care for this population and explore how pharmacists can better support newcomer international students and their families to facilitate their transition to a new healthcare environment.”*

Participants:-
Are the participants recruited from two institutions are generalizable about the situation of Canada?

We would like to thank the editor for raising this important question. Our proposed study aligns with the aims of qualitative research, which prioritize depth and detailed representation rather than generalizability. Therefore, our study focuses on the depth, richness, and complexity of understanding from a limited number of cases or participants.

- In response, we added to Discussion section: *“**Our goal is to deeply understand the specific experiences and challenges faced by the students who are part of the study, recognizing that these insights are representative of only those individuals' experiences. Thus, while the study provides valuable insights into the experiences of its participants, these findings are understood to be specific to the context and individuals studied, offering detailed understanding rather than broad applicability.”*

**Reviewer #1:**

1. Lines 56-111: How do new rules for international students in Canada affect this protocol/study?

We would like to thank the reviewer for highlighting this important point. To address this question, we added in the introduction section: *“While there are important benefits to furthering a diverse Canadian population that includes those new to Canada, rapid growth in newcomers has led to recent public policy changes to limit the number of newcomer international students as one mechanism to reduce national pressure on housing and everyday services including health care[14]. This measure reflects a deliberate shift towards a balanced approach, prioritizing both the well-being of international students and the capacity of local services to accommodate and integrate them effectively into Canadian society[14].”*

2. Lines 113-117: How is aim #1 different from aims #2 & 3? Please discuss.

- Aim #1 of the study focuses on the personal and subjective experiences of individuals as they navigate a new healthcare environment. It covers a wide range of experiences, including the initial understanding and adaptation to Canadian healthcare practices, cultural adjustments, and the emotional and psychological impacts of transitioning between healthcare systems.
- Aim #2, on the other hand, narrows the focus specifically to the challenges associated with medication use and management. This involves exploring specific issues such as understanding prescriptions, accessing medications, dealing with differences in medication availability or regulations, and managing ongoing treatments that were started in their home countries. This aim looks at practical and logistical aspects of medication management that international students and their families face in the Canadian context.
- Aim #3 extends the investigation into the structural and systemic aspects by identifying gaps in the healthcare delivery system that may affect the continuity of care for these newcomers. This could include gaps in service provision, barriers to access (like language barriers or lack of information), and inadequacies in policy that may disrupt the seamless continuation of healthcare services as students move from one healthcare system to another.

These details were not added into the paper because we also received a comment to ensure aims are concise.

3. Lines 123-125: Please provide a definition of “in-depth interviews”. Aren’t the interviews in a qualitative study already aim to explore ‘in-depth’ experiences of responders? What does the addition of “in-depth” mean here?

The addition of "in-depth" distinguishes these interviews from other qualitative methods like focus groups or structured interviews, where the scope for individual elaboration might be more limited due to the structured nature of questions or the group setting. In-depth interviews are particularly valuable in exploring complex issues, understanding personal stories, and gaining insight into contexts and behaviors that are not immediately observable. This method allows for a comprehensive exploration of the subject matter, which is essential in capturing the essence of participants' lived experiences and the meanings they attribute to them.

- To better define the “in-depth interviews”, we added in Study Design section: *“In-depth interviews aim at exploring comprehensive perspectives, experiences, and motivations through detailed, open-ended interactions between the researcher and participant. Additionally, it emphasizes deep understanding by using semi-structured interview guide with probing questions that encourage participants to extensively express their thoughts and feelings [28,29].”*

4. Lines 125-136: Thanks for providing references and explanations for “exploratory descriptive qualitative approach”. The qualitative approach is usually used to ‘explore’ and ‘describe’ phenomenon where there is little information available. Perhaps, authors should define what they mean by a “qualitative study” first for readership to understand the following sections about “exploratory” and “descriptive”.

Further, as authors indicate, the “descriptive … will go beyond simple observation and data recording…”. However, authors do not provide what are the qualifications of the state that will be achieved “beyond…”. Please discuss.

We agree with the reviewer, In response:

- We added in Study Design section: *“The qualitative approach will provide a deeper understanding of the healthcare transition experiences faced by newcomer international students and their families, focusing on the meanings and views of the participants[32].”*
- We added in Study Design section: *“This involves deeply engaging with the data to interpret and understand the implications and experiences of participants, thereby enhancing our understanding of how they experience healthcare transitions at both personal and community levels[31,33].”*

5. Lines 198-211: Could authors please discuss the types of guarantees (in terms of the privacy and confidentiality of interview data) that are provided by Microsoft Teams.

We would like to thank the reviewer for this important question. Microsoft Teams was approved as a secured platform to conduct and transcribe the study interviews by the University of Toronto Health Sciences Research Ethics Board (REB # 44770). Interview transcripts will be stored electronically on a secure network using SharePoint on the University of Toronto server and all recordings will be permanently deleted once the analysis is complete.

- We added in Data Collection Procedures section: *“The generated transcripts will be reviewed, de-identified, and stored electronically on a secure network affiliated with the university. Any personal identifiers (names of individuals, locations, organizations, etc.) will be removed from the transcripts. The interview recordings will be securely stored and kept until transcription and analysis are completed. Once these processes are finalized, all the recordings will be permanently deleted to ensure participants' privacy and confidentiality are maintained in accordance with ethical research guidelines.”*

6. Lines 208-210: Please discuss if authors have concerns about how comparable the interview responses will be when camera is on as opposed to when camera is off.

We would like to thank the reviewer for raising this critical point. We want to confirm that we do not have concerns from a methodology perspective. Our study employs conventional content analysis, which primarily focuses on the data extracted from transcripts and audio recordings. This approach is distinct from discourse analysis, where non-verbal cues, such as body language, play a crucial role in data interpretation. Therefore, the comparability of interview responses is not significantly impacted by whether the camera is on or off during the interview. Thus, allowing participants the option to turn off their cameras aims to enhance their comfort without compromising the integrity and depth of the data collected for content analysis. This flexibility helps ensure that participants feel at ease during the interview, potentially leading to more open and comprehensive responses, which are vital for the richness of the data in conventional content analysis.

7. Lines 213-231: Who will conduct the interviews? Later, it is reported that there will be at least one interviewer (line 365). Please discuss if there will be other interviewers.

We would like to confirm that only one interviewer will conduct the study interviews.

8. Lines 213-231: Will there be debriefing after interviews? Authors do discuss revising the guide later but that seems to be only tied to the data analysis. Please specify.

We would like to confirm that there will be no debriefing after the interviews. The semi-structured interview guide will only be revised and updated based on the responses to the interview questions.

- To clear the raised confusion regarding updating the interview guide, we added to Data Analysis and Reporting section: *“Updating the interview guide will involve only minor changes, such as including additional probes or modifying the language of some questions if they are found to be misunderstood.”*

9. Lines 233-243: Will the interview guide be pilot tested?

We agree with the reviewer that the interview guide should be tested. In response:

- We added to Interview Questions section: *“**To ensure the clarity and effectiveness of these questions, the interview guide will be pilot tested with a small group of participants (2-3). This pilot test will help refine the questions to better capture comprehensive and relevant data.”*

10. Lines 234-237: “Semi-structured” interviews? Please tie back to “in-depth” interviews.

We would like to clarify that in-depth interviews will follow a semi-structured interview guide that consists of 5 open-ended main and probing questions. Thus, ‘semi-structured’ refers to the guide not the interviews.

- To address the highlighted confusion, we added in Study Design section: *“In-depth interviews aim at exploring comprehensive perspectives, experiences, and motivations through detailed, open-ended interactions between the researcher and participant. Additionally, it emphasizes deep understanding by using semi-structured interview guide with probing questions that encourage participants to extensively express their thoughts and feelings [28,29].”*

11. Lines 245-272: How will authors ensure that their results are credible?

We would like to thank the reviewer for pointing out this critical point. To ensure the results credibility in our study:

- The findings will be presented using several quotes from different participants. This approach not only increases transparency but also allows readers to see the evidence base for the researchers' interpretations, enhancing the credibility of the data.
- We will utilize the COREQ (Consolidated Criteria for Reporting Qualitative Research) checklist throughout the study. This is a widely recognized framework that ensures high-quality reporting of qualitative research findings. Adhering to COREQ standards helps in increasing the trustworthiness of the study and promotes explicit and comprehensive reporting of the methodology and findings. As detailed in Data Analysis and Reporting section: *“The Consolidated Criteria for Reporting Qualitative Research (COREQ) checklist will be used as an integrated framework for reporting the study as it is suitable for studies using interviews. Using the COREQ checklist will ensure high-quality reporting of findings, increase the trustworthiness, and promote explicit and comprehensive reporting of the study. Additionally, it will cover the important role of researchers in the qualitative study by adding the context of their interpretation of the study findings and the assumptions they may have made. This will lead to increasing the study’s transparency.”*

In addition, a second coder will be involved in the data analysis phase to produce rich data and minimize bias. We added in Data Analysis and Reporting section: *“The coding will be conducted by two researchers, each will be working separately on a subset of the interview transcripts. This dual-coding approach is essential for assessing the consistency between their analyses, ensuring that the analysis is unbiased and mutually exclusive, and enriching the data quality.”*

12. Lines 245-255: Who will do the coding and other data analyses and how? There seems to be one interviewer (line365). Does that mean there will be one coder? Please describe the process clearly.

In response to the reviewer’s question, we added to Data Analysis and Reporting section: *“The coding will be conducted by two researchers, each will be working separately on a subset of the interview transcripts. This dual-coding approach is essential for assessing the consistency between their analyses, ensuring that the analysis is unbiased and mutually exclusive, and enriching the data quality.”*

13. Lines 264-272: “COREQ”? Thanks for providing a reference but please ensure that the acronym is written out the first time it is mentioned.

We agree with the reviewer’s comment. In response, we added to Data Analysis and Reporting section: *“The Consolidated Criteria for Reporting Qualitative Research (COREQ) checklist will be used as an integrated framework for planning and reporting the study.”*

14. Lines 288-294: Will there be any services available for those international students who become emotional during interviews?

We would like to thank the reviewer for highlighting this point. We added to Ethical Considerations section: *“**Additionally, while we are unable to provide direct therapeutic services, we will offer a list of accessible mental health resources and support services available to them as international students at U of T, ensuring they have options to seek help if needed. This approach ensures the ethical handling of emotional responses while respecting the participants' emotional well-being.”*

15. Line 298: Again, “explorative descriptive qualitative study”?

We deleted the redundant sentence.

16. Lines 323-327: Could authors explore which countries (of that total 200) are represented by U of T international students rather than reporting this as a limitation?

We would like to thank the reviewer for this question. To response to this, we add to Discussion section: *“U of T continuously welcomes international students from a diverse array of countries. According to the latest information provided by the university, more than 25% of the student body is international, coming from 170 different countries[47,48].* *The study sample includes approximately 20 participants. Consequently, our study emphasizes detailed understanding from these participants rather than broad applicability to all international students at U of T. The findings may therefore reflect the experiences of international students from only the represented foreign countries.”*

17. Lines 323-324: Please refrain from discussions that resemble (or make the reader think of) generalizability and give the impression that this protocol is for a quantitative study.

We agree with the reviewer. In response, we edited in Discussion section: *“Hence, the study findings may only reflect the experiences of international students from the represented foreign countries and might not capture the challenges associated with transitioning from other countries not included in the study sample.”*

18. Lines 324-327: Again, which countries are those? Further, even if U of T had international students from all of those 200 countries, which characteristics of a qualitative study provide the researcher with the tools that will make the generalizability of their findings possible? Please discuss.

We would like to thank the reviewer for raising this important question. Our proposed study aligns with the aims of qualitative research, which prioritize depth and detailed representation rather than generalizability. Therefore, our study focuses on depth, richness, and complexity of understanding from a limited number of cases or participants. Our goal is to deeply understand the specific experiences and challenges faced by the students who are part of the study, recognizing that these insights are representative of only those individuals' experiences. Thus, while the study provides valuable insights into the experiences of its participants, these findings are understood to be specific to the context and individuals studied, offering detailed understanding rather than broad applicability.

**Reviewer #2:**

1. what types of challenges are expected to be identified?

In response to the reviewer’s question, we added to Abstract*: “….. their main challenges related to developing coping skills for managing diseases and navigating the complexities of prescription and nonprescription medication use.”*

2. Do you mean the research will be conducted among the international students only? What about their family? Specify whether the study will focus solely on international students or also include their families. If the study includes both groups, it should be explicitly stated in the abstract. If it is not included then its better to think

We would like to thank the reviewer for this comment. To clarify, our study will only include conducting interviews with newcomer international students. However, these students, particularly those who have arrived in Canada with their families, will also provide insights into the challenges and healthcare needs their families have encountered, helping to inform broader healthcare solutions.

3. Can you elaborate on the specific actions that need to be modified by the pharmacist for the better services?

To address the highlighted point, we added to the Abstract: *“By exploring the healthcare challenges faced by these students, our findings will help identify areas where pharmacists can practice to their full scope including in medication management, patient education, and transitional care. Consequently, the study will offer detailed recommendations on how pharmacists can better support this population and thereby help alleviate the overall strain on the primary healthcare system.”*

4. Better to explore about the challenges faced by the international students and their family regarding pharmaceutical services and its impact on the role of pharmacist and how to tackle such challenges to foster the healthcare services.

We would like to thank the reviewer for pointing out this important point. To clarify, we aim to explore the diverse challenges faced by newcomer international students and their families as they transition to the Canadian healthcare system, particularly focusing on primary healthcare system as well as pharmaceutical care services. This exploration will help us identify gaps in the current healthcare system and determine how pharmacists can better support this population through enhanced improved pharmaceutical care, education, and referrals to appropriate healthcare services. As detailed in our manuscript, the full scope of pharmacy practice is well aligned for meeting the diverse healthcare needs of newcomer international students, especially when they are not registered with a family physician and may have misconceptions or lack accurate information about the available healthcare services in Canada. This approach will allow us to address how pharmacists can effectively tackle these challenges to improve the overall delivery of healthcare services to this group.

5. Please make the objectives clear, concise, and readers friendly. Clearly stating the research questions or objectives would help redear understand the study’s purpose more clearly.

In response to the reviewer and editor’s comments, we restructured the study's Aims and Objectives: *“Our study aims to provide a comprehensive understanding of the unique lived experiences of international students. We will explore the healthcare needs of these students as they transition from healthcare systems in their countries of origin to Canada. Additionally, the study will identify the diverse challenges that these students face. It also seeks to uncover gaps in healthcare delivery that may hinder the continuity of care for this population and explore how pharmacists can better support newcomer international students and their families to facilitate their transition to a new healthcare environment.”*

6. I think it’s a recommendation thing that will have different section so I don’t think its better idea to make it a objectives.

We agree with the reviewer’s comment. In response, we deleted this section in Aims and Objectives.

7. May I know the specific rationale for choosing participants who have been in Canada for more than one month but less than five years. Is there a particular reason for this time frame in relation to their healthcare transition experiences?

We would like to thank the reviewer for raising this important question. In response, we added to Sampling Strategy section: *“This timeframe is selected because newcomers are defined as those who have been in Canada for less than five years[3]. In addition, setting a minimum time of one month helps ensure that participants have had sufficient time to engage with the healthcare system, allowing them to reflect on their initial experiences and the challenges faced during their early adaptation period.”*

8. Are there any specific measures taken to ensure participant understanding of the consent form, especially for participants whose first language may not be English??

We would like to thank the reviewer for this question. To clarify, the study participants include international students at the University of Toronto who can speak English, as detailed in the study inclusion criteria.

- Additionally, to address the highlighted point, we added in Informed Consent section: *“In addition, to accommodate participants whose first language is not English, the research team will address any language-specific questions or concerns to clear up any confusion and ensure a comprehensive understanding of the consent process before conducting the interview.”*

9. How do you ensure that the REDCap link for viewing and signing the consent form is easily accessible and user-friendly, especially for participants who may not be familiar with technology or online platforms? Is there any alternative available to those who cannot access it and sign over the link?

In response to the reviewer’s question, we added to Informed Consent section: *“Alternatively, a copy of the study consent form will be provided to participants by email or mail according to their preference. The participant can return the signed hard copy by email or mail in a prepaid envelope included in the participant consent form package.”*

10. Can you elaborate more on it?

We added more details on the consent for review process in Informed Consent section: *“**This review process will include a thorough explanation of the study's purpose, procedures, and participant rights to ensure complete understanding. Participants will have the opportunity to ask questions and seek clarifications, allowing them to make an informed decision about their involvement. This step is crucial for maintaining ethical standards and ensuring that all participants are fully aware of what participation entails.”*

11. Is this voluntary participation or not? How do you ensure the confidentiality and privacy of the participants and how you establish their trust in it?

We would like to thank the reviewer for raising this question. To clarify, as per the guidelines of the University of Toronto Health Sciences Research Ethics Board (REB), the study consent form includes sections that cover detailed information about participants’ Privacy and Confidentiality as well as Voluntary Participation. The study consent form was approved by REB (REB # 44770). Moreover, we added more detailed information to address this point as follows:

- We added in the Informed Consent section: *“The consent form clearly states that participation in the study is completely voluntary.”*
- We added in Data Collection Procedures section: *“The generated transcripts will be reviewed, de-identified, and stored electronically on a secure network affiliated with the university. Any personal identifiers (names of individuals, locations, organizations, etc.) will be removed from the transcripts. The interview recordings will be securely stored and kept until transcription and analysis are completed. Once these processes are finalized, all the recordings will be permanently deleted to ensure participants' privacy and confidentiality are maintained in accordance with ethical research guidelines.”*

12. Does it mean the company will provide them a place to stay for one hour in a private and give a interview? Please elaborate this process as it’s a virtual interview. You need to ensure the background of the participants to talk to you freely for a valid and reliable answer.

We would like to thank the reviewer for this important point. To clarify, all students at University of Toronto including international students have access to private quiet spaces including the libraries for personal, professional, and academic purposes which they are informed of as a part of their admission package. <https://onesearch.library.utoronto.ca/study-spaces-and-carrels>

- To address this comment, we also added to Interview Method section*: “* *Participants will select their preferred location for conducting the virtual interview.”*

13. What do you mean by effectiveness over here? And how participants will be asked to evaluate the effectiveness of the services they have used??

In response to the reviewer’s question, we edited this part to be more detailed in Interview Questions section: *“In addition, participants will be asked to describe their experiences with the current healthcare services they have been using to manage their health conditions. Specifically, they will be prompted to discuss their perceptions of how these services have met their healthcare needs, including any improvements in their conditions and satisfaction with the service received.”*

14. Is there any reference regarding this? Please reference it.

We added the required references.

15. Are there any plans to securely delete and dispose the recordings once they are no longer needed?

In response to the reviewer’s question, we added in Data Collection Procedures section: *“The generated transcripts will be reviewed, de-identified, and stored electronically on a secure network affiliated with the university. Any personal identifiers (names of individuals, locations, organizations, etc.) will be removed from the transcripts. The interview recordings will be securely stored and kept until transcription and analysis are completed. Once these processes are finalized, all the recordings will be permanently deleted to ensure participants' privacy and confidentiality are maintained in accordance with ethical research guidelines.”*

16. Are there any measures that will be taken to mitigate potential emotional distress experienced by participants during interview and how will the data be handled if the participants withdraw the interview at the middle?

We would like to thank the reviewer for raising this important question. In response, we added more information to address the highlighted point as follows:

- We added to Ethical Considerations section: *“Additionally, while we are unable to provide direct therapeutic services, we will offer a list of accessible mental health resources and support services available to them as international students at U of T, ensuring they have options to seek help if needed. This approach ensures the ethical handling of emotional responses while respecting the participants' emotional well-being.”*
- We added to Ethical Considerations section: *“If a participant chooses to withdraw from the study, any collected data will be discarded upon their request.”*

17. Please provide more references for the discussion.

We added more references to the Discussion section.

**Reviewer #3:** This is an excellent and novel protocol that holds promise in shedding light on an important and under-researched topic in Canada. The methods are clearly described, and the rationale for the study is also clear. I have only three main comments for consideration.

First, though the motivation for your study is about the issues facing newcomers in terms of access to care and inequities in health outcomes, the focus on international students may limit the ability to generate insights into the challenges facing newcomers more broadly, especially those in more structurally disadvantaged positions. Indeed, international students may be among the more privileged and may have advantages often described as the healthy immigrant effect, whereby levels of health on average for this population are better than for average Canadians, or newcomers more broadly. Could the authors elaborate on the importance of this particular subgroup of the newcomers population and how insights gained from this study could still be informative in terms of the broader issues presented in the introduction?

We would like to thank the reviewer for this comment. Certainly, our focus on international students does indeed represent a subgroup that may not entirely capture the broader experiences of all newcomers, particularly those facing more significant structural disadvantages.

However, this specific focus is both strategic and significant for several reasons that we added to Discussion section to address this comment: *“**International students, while often perceived as privileged, face unique challenges and stressors related to academic pressures, cultural adaptation, and in some cases, the complexities of navigating a foreign healthcare system[39,45]. These factors can contribute to specific health vulnerabilities that differ from those of other newcomer groups and the general population[12,15,39]. The insights gained from studying this particular subgroup can provide valuable information on the integration processes and the effectiveness of current healthcare services tailored to address the healthcare needs of non-permanent residents in Canada[38]. Understanding these aspects can help identify gaps in services and inform policies that enhance healthcare accessibility and equity not only for international students but also for other newcomer groups. International students often form a bridge between their communities and the host country, influencing both cultural exchange and economic ties[15]. Studying their healthcare experiences can thus offer broader insights into how health systems and policies affect an internationally mobile population, which can have implications for global health policies and international collaboration[46]. Therefore, while the study concentrates on international students, the findings can illuminate aspects of the healthcare system's functionality and responsiveness, offering perspectives that are applicable to the broader discussion on healthcare accessibility and equity for all newcomers[46]. This approach allows for a focused investigation that can still contribute to understanding and addressing the wider challenges faced by newcomers.”*

Second, it would help to clarify the recruitment a bit more, as it strikes me that the use of a pharmacy to recruit international students may select only those who already have some prior knowledge of existing care options available to them and who are able to access it vs those who are less aware of on campus services and supports and therefore may be facing greater barriers to care that are worth uncovering in the study. It would be great if the authors could comment on this potential limitation, or correct my misunderstanding.

We would like to thank the reviewer for providing this comment regarding the recruitment strategy. The recruitment of newcomer international students through Discovery Pharmacy, an academic not-for-profit community pharmacy affiliated with the University of Toronto (U of T), is indeed one pathway to engage students who may have already started accessing healthcare services. This recruitment method can provide valuable insights into the experiences of those who have some level of familiarity with available healthcare options. However, to ensure a broader perspective that includes those who may not be as aware of or able to access existing healthcare services, we are also employing additional recruitment methods. As detailed in Recruitment section in our manuscript, alongside the pharmacy-based recruitment, we are using study posters and leveraging formal and informal networks on campus, including international students’ advocacy groups and social media platforms. This dual-faceted approach aims to reach a wider array of participants, including those who may face greater barriers to care and who might not frequent the pharmacy.

- To address the highlighted possible confusion, we added to Recruitment section: *“**By diversifying our recruitment strategies, we aim to gather a more comprehensive range of experiences from international students, encompassing both those who are engaged with existing healthcare services and those who may be less integrated. This will enhance our study's capacity to capture a diverse array of challenges and experiences related to healthcare transitions among newcomer international students at U of T.”*

Third, I understand that the motivation for this study is to examine the potential role of pharmacists in assisting navigation and transition to Canada's health systems for newcomers, but is there also room to uncover opportunities for other health providers, and perhaps gain insights into the opportunities to improve access or quality of care for this population by other care providers, such as primary care physicians, nurses, walk-in clinics, mental health services, etc? The broader implications of your study could be considered in the Discussion section, since the open-ended nature of the questions asked in the interview guide may yield new insights into these other aspects of health system.

We would like to thank the reviewer for raising this important point. While the primary focus of our study is on the role of pharmacists in assisting newcomer international students with navigating Canada's healthcare systems, we agree that we can gain valuable insights that help explore the potential roles of other healthcare providers in supporting newcomer international students as they transition to the Canadian healthcare system.

- In response, we added to Discussion section: *“Additionally, the findings from this research could reveal broader opportunities for improving healthcare access and quality, not just through pharmacists but through a more integrated approach involving other healthcare professionals[25]. Understanding how international students navigate healthcare challenges could lead to improvements in service delivery across a variety of healthcare settings, including primary care facilities and mental health services[45]. This could include better coordination between healthcare disciplines, tailored support systems for international students, and facilitated access to essential services, ensuring that all healthcare providers are better equipped to meet the diverse needs of this group effectively.”*
